# Multiple subject tracking in long-term, behavioral studies of rodents

**DOI:** 10.3389/fnmol.2026.1960212

**Published:** 2026-09-22

**Authors:** Bartosz Zglinicki

**Affiliations:** Functional Ultrasound Imaging on Brain Circuits Research Group, P4Health – Centre of Excellence for Precise Phenotyping and BioDataBanking, Łukasiewicz – PORT Polish Center for Technology Development, Łukasiewicz Research Network, Wrocław, Poland

**Keywords:** arUco, automation, behavior, color, identity, pose estimation, RFID, tracking

## Abstract

High-resolution, automated or semi-automated behavioral phenotyping of rodents is increasingly used in neuroscience to reveal behavioral characteristics, that are unreachable with standard manual methods. In fact, these computational approaches have become so remarkable in recent years, it is possible now to extract not only basic ethograms of animals in an observable environment, but also to dissect individual components of particular behaviors. Currently, most computational phenotyping is based on image/video processing. The automation of such behavioral analysis promises consistent, high-throughput, unbiased results, and in general sense that can be true. Nevertheless, when looking more closly at the methods’ workflow and its implementation, some obstacles inevitably emerge. In this article, the issue of identity mismatches in multi-animal, behavioral studies will be discussed, which is especially significant for long-term (multi-hour, multi-day) format. It will briefly explain how in image-based, machine learning approaches, the identity of a subject can be maintained and why algorithms struggle to do so in some situations. In response to those limitations, additional identity-tracking solutions will be presented (ArUco tags, RFiD tags, and color-based recognition) that may significantly improve the overall process of behavioral screening that relies on available open-source tools for pose estimation, such as DeepLabCut or SLEAP.ai.

## 1 Introduction

The importance of animal behavioral studies in neuroscience cannot be undermined. Behavior is an observable manifestation of a functioning nervous system, providing insights into the actions performed by a subject within an environment. Scientifically conducted behavioral studies on animals can be traced back as far as the 1860s (Gray, 1967), however, attempts of long-term, multi-animal behavioral observations of rodents appeared later on Calhoun (1973), Calhoun (1950), Crowcroft (1955), and Marsden (1972). Long-term studies can offer significant advantages in data volume and data complexity over well-established, short-term behavioral tests many scientists use today. These long-term studies can be quite demanding to establish/conduct however. In the past it required manpower and manpower reliability during monitoring, as all relevant events and individual animals had to be meticulously supervised manually. Currently, with accelerating pace of technological development, manpower can be significantly reduced, but reliability of technology (and reliability in using the technology) is still important. High-resolution, automated or semi-automated behavioral phenotyping of rodents is increasingly used in neuroscience to reveal behavioral characteristics that are unreachable with standard manual methods (Bordes et al., 2023; de Chaumont et al., 2019; Forkosh et al., 2019; Nagy et al., 2024; Peleh et al., 2019; Puścian et al., 2016). In fact, these computational approaches have become so remarkable that it is now possible to extract not only basic ethograms of animals in an observable environment, but also to dissect individual components of particular behavior (Bordes et al., 2023; Hsu and Yttri, 2021; Lin et al., 2024; Weinreb et al., 2024). Currently, most computational phenotyping is based on image/video processing, although audio-based approaches should be mentioned here, as vocalization is inherently tied to rodent behavior. For anyone interested in behavioral phenotyping based on image processing, there are multiple, densely packed articles that describe a vast number of methods available, and steps needed to design, conduct, and analyze the experiment of choice (Jurek et al., 2026; Mathis and Mathis, 2026). Current automation of behavioral studies promises consistent, high-throughput, unmatched results and in general sense that can be true. Nevertheless, when looking more closely at the method’s workflow and its implementation, some obstacles inevitably emerge and have to be addressed to ensure the registration of true positive events. The aim of this article is to emphasize the issue of subject identity maintenance during long-term, semi-automated monitoring, where multiple animals are placed and observed within a shared environment. The article will explain where identity issues may arise from and what additional identity-tracking solutions can be incorporated in order to improve the overall process of behavioral screening that relies on available open-source tools for pose estimation, such as DeepLabCut (Lauer et al., 2022) or SLEAP.ai (Pereira et al., 2022).

## 2 Maintaining subject identity

Every system designated for multi-animal behavioral screening has to assign unique identities to subjects and maintain them throughout the whole experiment. Failure to do so may result in improper distribution of detected behavioral events among the respective animals. The following are the most commonly used approaches that make subject tracking possible.

### 2.1 Markerless approach

Development of markerless, image-based computational methods of subject tracking peaked with machine learning-based detection and the implementation of neural networks. Its popular end result – pose estimation – is now very frequently used in behavioral studies (Graving et al., 2019; Karashchuk et al., 2021; Lauer et al., 2022; Pereira et al., 2022). It allows for both subject tracking as well as body parts (keypoints) tracking and is appealing because it ultimately opens up numerous possibilities for interpreting this tracking data into meaningful, behavioral findings.

#### 2.1.1 Working principle

Pose estimation tools assign and maintain animal identity in a step that is separate from body parts prediction. Object tracking and keypoints tracking are associated with each other however, and this association is characterized by either a bottom-up or a top-down approach within pose estimation software. The top-down approach is generally recommended for behavioral studies of rodents and therefore will be a subject of discussion here. In the top-down approach, the software establishes bounding boxes (bboxes), rectangular borders surrounding each of the animals (Figure 1A). These borders are assigned based on researcher input during the annotation process (manual keypoints placement) and subsequent inference (prediction) of all body parts. Afterwards, a tracker function will try to assign and match unique identities by comparing animals’ bboxes frame by frame with a specific cost function that utilizes characteristics of bboxes (more details in Figure 1B). In the literature, even more specific and advanced details can be found regarding bounding box comparison with similarity measures (Rezatofighi et al., 2019; Nou et al., 2024; Su et al., 2024; Zheng et al., 2020), cost functions, or with additional optimization algorithms (Xiao et al., 2018; Redmon et al., 2016; Kalman, 1960; Kuhn, 1955). The purpose here is to describe the matching technique in a sufficient way to have an understanding of why this method fails in some situations and leads to identity mismatching.

**Figure 1:**
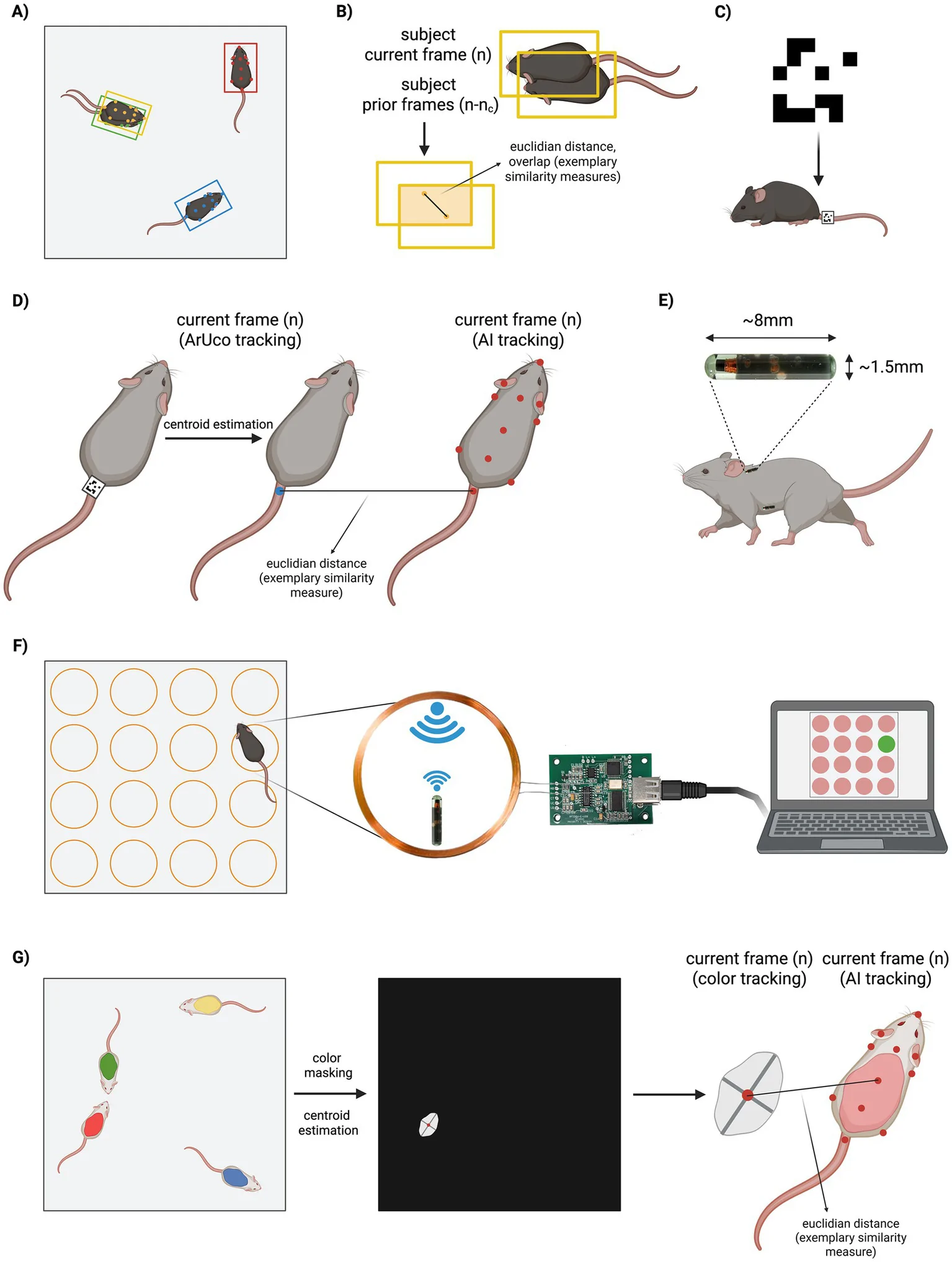
General principles of automated subject tracking in behavioral studies. **(A)** An exemplary open field-type arena with four mice inside, highlighted with their respective pose estimation keypoints and bounding boxes. Keypoints and bboxes are marked with colors here to differentiate between subjects. Red and blue mice are easy to distinguish in that configuration, as their bounding boxes are far apart. Green and yellow mice may pose difficulty in maintaining their stable identities, as their bounding boxes are very close to each other and overlap significantly. This is more problematic the longer they stay together. **(B)** With bounding box implementation (marked in yellow), identity of a subject is maintained by comparison of bboxes of the current frame (*n*) and immediate prior candidate frames (*n*-*nc*). Candidates (*nc*) can consist of only one frame or multiple frames, with the latter one having possibly higher chance of improving tracking efficiency. Exemplary features like euclidian distance between centroids of bboxes or their overlap can be used for comparison. The smallest the euclidian distance and the highest the overlap, the bigger the chance that adjacent bounding boxes belongs to the same subject. This is also a reason why animals that stay very close to each other for longer periods of time are prone to being mismatched. **(C)** Tracking with fiducial (ArUco) method involves attachment of a tag onto the subject, ensuring constant availability of the code for a designated camera to scan it (Coulombe et al., 2025). **(D)** Tracking with fiducial (ArUco) method involves a video framerate-dependent, high throughput code scanning for identity retrieval and a tag centroid placement. Continuous tracking of centroid position can serve as an identity reference and be used for potential correction of mismatched identities, delivered with AI tools. Potential correction can be applied by calculation of the exemplary euclidian distances between ArUco centroid and AI-derived corresponding keypoints of all subjects from the same current frame (*n*). The shortest distance should correspond to the same animal. If it is not, AI-derived identity can be overwritten with ArUco’s referenced identity. **(E)** Tracking with RFiD technology requires transponder placement in a body of the animal, usually subcutaneously to ensure minimal proximity to a reader. Areas around the neck and abdomen are commonly chosen. **(F)** An exemplary open field-type arena with circular, evenly spaced readers’ antennas (based on de Chaumont et al., 2019). Antennas have constant current flowing in them under the control of the board (for simplicity only one control board is presented, in reality each antenna has its own board and all of them may connect through USB-hub into the computer). If an animal equipped with RFiD tag reaches its range, the identity signal is transmitted and registered by the control board and computer software. **(G)** An exemplary open field-type arena with painted mice available for color tracking. For each color an appropriate threshold is applied in a separate layer (for simplicity only red color layer is shown). A centroid is then calculated and applied. Continuous tracking of centroid position can serve as an identity reference in a similar manner as with previous methods, with euclidian distance measurements being used to correct any potential identity mismatches. Created with BioRender.com (https://BioRender.com/got9sta).

#### 2.1.2 Limitations and challenges

Tracking algorithms in pose estimation tools fail most of the time during instances where subjects are in very close proximity to each other for a prolonged time. Common examples are animals moving over or under each other frequently or sitting next to each other while resting, sleeping, eating, etc. In these instances, animals’ bboxes may become computationally indistinguishable, making correct identity assignment very difficult. To address this issue, pose estimation programs offer tools for manual correction of mismatched identities, where users can pinpoint the exact frames at which errors occurred and fix them accordingly. This process is called proofreading. Unfortunately, for long-term studies (multi-hours, multi-days), proofreading can be particularly tedious and require long hours to carry out. Markerless approaches are also not suited for studies that introduce a heavily enriched environment. Small amount of cotton rolls or wooden blocks may not be a problem, but any additions that substantially occlude animals will cause major issues.

#### 2.1.3 Advantages and recommendations

Proofreading is very useful when screening relatively short videos (i.e., 10–60 min in duration). In this case, the process should not be overly time-consuming and will provide great efficacy. Moreover, if short-term behavioral testing is performed in a fresh, novel environment, this often triggers exploratory behavior (Chrzanowska et al., 2022; Mazurkiewicz et al., 2022; Thompson et al., 2018), where animals are scattered around the arena and not preoccupied much with each other. This outcome does not introduce many challenging instances for tracker functions to differentiate between subjects. For multi-hour and multi-day recordings however, implementation of external identity markers is highly recommended. Researchers also have to remember that working with pose estimation tools is computationally demanding. A solid graphics card (GPU), such as the Nvidia RTX series, is going to be advantageous and will significantly decrease the time that is necessary for training the model and predicting both animal body parts and identities.

### 2.2 Fiducial/ArUco tags

This method was already utilized before the rapid rise of pose estimation technology (Crall et al., 2015), nevertheless, it is used currently in the behavioral research of rodents as well (Coulombe et al., 2025).

#### 2.2.1 Working principle

Fiducial tags/markers resemble “quick-response” (QR) codes and share the same working concept (Figure 1C). They can link their unique signature to specific information, in this case – animal identity. The code has to be attached to a subject and be visible to the camera. Tracking with the ArUco method involves a video framerate-dependent, high-throughput code scanning for identity retrieval. Detection of markers is carried out with a freely available OpenCV-based algorithm (Garrido-Jurado et al., 2014), that can be controlled through numerous programming languages (i.e., python-based scripting), making it accessible for prototyping and “do it yourself” (DIY) implementations.

#### 2.2.2 Limitations and challenges

Just as with any other image-based processing method, ArUco tracking is sensitive to major occlusions. Therefore, it is not suited for studies that introduce a heavily enriched environment.

#### 2.2.3 Advantages and recommendations

In instances where constant and clear visibility is ensured, fiducial tags will be a good subject tracking implementation for behavioral studies. Just like with QR codes, it is enough for the fiducial tags to be black and white in colors, which means they can be scanned with monochrome cameras and are suited for recordings under infrared (IR) light. This is particularly useful for nocturnal animals, such as rodents. Their peak activity, and hence broad behavioral repertoire, falls within periods of darkness, and this is when running trials is recommended (Hawkins and Golledge, 2018). Lighting condition flexibility allows these tags to be well adapted for long-term monitoring, in which tag readings may occur in both night and day periods. These markers can be scanned well up to several dozen frames per second (FPS), which is enough for the majority of video monitoring in behavioral research. Even for fast-paced behavioral events (requiring much higher framerates), more specialized tracking libraries are also available (Garrido-Jurado et al., 2026; Ulrich et al., 2018). Generally, ArUco libraries will not be computationally demanding and can be launched on standard computers, although the latest CPUs and higher amounts of memory would be beneficial for big animal cohorts within camera reach. Ultimately, this method of subject tracking can greatly support markerless approaches and their own limitations. Whenever experimental footage is processed with a pose estimation tool, any identity mismatches can be supported and corrected with ArUco subject tracking data by applying similarity measures (Figure 1D).

### 2.3 RFiD tags

Radio Frequency Identification (RFiD) technology has been around for decades. The concept was first introduced in the 1930s for military purposes (Trim, 2003), but the method found application in many industrial, medical, and scientific areas, including animal behavior studies (de Chaumont et al., 2019; Fong et al., 2023; Kiryk et al., 2020; Peleh et al., 2019; Puścian et al., 2016).

#### 2.3.1 Working principle

The technology uses radio waves to transfer data between a tag (transponder) and a reader and generally does not need any video camera input. However, hybrid systems do exist and combine image-based approaches with RFiD technology. Although RFiD tags appear in passive and active variants (an active one includes battery power for long-range detection), in relatively small enclosures, such as open field-type boxes, the passive variants are generally used. An individual tag contains a tiny microchip, which can store identity information, and a tiny antenna connected to the chip itself. The antenna is necessary to establish a connection with the reader and harvest energy from it to function properly. Readers, however, are more substantial and contain a control board connected to a much larger antenna then the one inside a transponder. Antennas are just copper wires shaped in different forms and positioned accordingly to ensure the most efficient availability for the transponders to catch their signal. The signal is enabled by a current that flows through the antenna, regulated by the control board. Tracking with RFiD requires placement of a transponders in a body of animals (Figure 1E). Whenever the RFiD tag reaches the electromagnetic field of the antenna’s reader, it sends the information encoded within its microchip (i.e., identity signature) (Figure 1F). The number of readers depends on the overall design of the system, its purpose, and desirable tracking resolution. For example, in a commercially available IntelliCage system, intended for cognitive screening, they are placed at the top of the entrance to the learning corners (one reader per corner) and scan any mouse that moves into/out of it. In non-commercial, open field-type systems, such as the popular Live Mouse Tracker, the antennas (circular copper coils) of the readers are placed and evenly spaced just below the floor to guarantee coverage of the whole arena and thus consistent tracking (Figure 1F).

#### 2.3.2 Limitations and challenges

Tracking with RFiD does rely on solid communication between tags and readers. If they are too far apart, the signal can be lost, leading to missing tracking data. A good example is too much bedding in the arena, or instances where an animal climbs high on top of included toys, ramps, etc., in setups where reader antennas are located below floor level. RFiD technology is also more challenging to incorporate into DIY setups than other methods presented here. In terms of hardware, aside from a standard computer and a camera, it requires multiple readers, transponders, antennas, USB hubs, connection cables, and soldering skills to make a functional tool. The computational aspect of RFiD subject tracking is crucial to address as well. Although the method is not very demanding in terms of processing power, estimating subject position is not always straightforward. The location of the transmitted signal between a tag and a reader can be quite general and related to the space occupied by the antenna (a tag may arrive at the coil electromagnetic field from multiple directions). It might not provide a precise location of a tag on its own. This is fine for some experimental designs (e.g., registering animals moving through a corridor), but is not sufficient for continuous, precise monitoring in open arenas. For that purpose, additional calculations are needed and incorporate parameters such as signal strength, time of arrival, and angle of arrival that must be additionally provided (Li et al., 2009).

#### 2.3.3 Advantages and recommendations

The biggest perk of RFiD technology is its working principle, which allows for conducting experiments with a substantially enriched environment. In fact, it might be the most suitable method for efficient subject tracking under such requirements. But even without enrichment, RFiD can still be incorporated as a valuable tracker, alone or as a support in hybrid systems, alongside primary image-based processing (Fong et al., 2023; de Chaumont et al., 2019). Successful computation of animal positions (centroids) with RFiD can be used to repair identity mismatches introduced by image-based processing by applying similarity measures. Alternatively, RFiD readers can function as checkpoints where potential identity errors can be corrected. During implementation of RFiD tracking, there is a fair amount of chance that for a designated behavioral study, a researcher will have to use multiple readers in order to correctly track subjects. For any behavioral, DIY implementation, the number of readers has to be thoughtfully chosen to balance costs, sufficient tracking resolution, and necessary component usage. An important factor here is also the relationship between a transponder’s size and the reader antenna’s size. Generally, the bigger the antenna (in both transponder and reader), the longer the communication range. This also has to be carefully considered and will be inherently tied to an animal species (a rat will definitely require bigger antenna components than a mouse). Transponder placement inside the body of an animal is rather intuitive and should guarantee its readability by readers’ antennas. Subcutaneous placement in the abdomen or neck is common (Figure 1E) and requires a surgical procedure or the use of dedicated transponder injectors. Both approaches should be carried out under anesthesia.

### 2.4 Color-based identification

Possibly one of the most overlooked method of animal identity tracking in behavioral studies is color-based recognition. It does not necessarily mean the natural color of a particular species, but rather the external application of color. Especially in cases of rodents, this can spark some interest, as under certain circumstances it can be the easiest tracking method to implement.

#### 2.4.1 Working principle

This method relies on applying colored paint or dye directly to the fur of animals (Forkosh et al., 2019; Nagy et al., 2024). Individual colors can then be independently recognized even by a single camera with fairly simple image processing. For each of the selected dyes, appropriate thresholding has to be applied to the video image in a separate tracking layer, so that everything except the color of interest is disregarded by the camera (Figure 1G). For the single body area that is detected and represents the color (a “blob”), a centroid can be calculated and applied, ultimately allowing for precise tracking of a subject through the whole video. This tracking output can serve as a reference for repairing any occurring subject mismatches, delivered with pose estimation programs if they were used. The correction is carried out in a similar manner as with arUco or RFiD methods (Figure 1G).

#### 2.4.2 Limitations and challenges

The biggest limitation of that method is the animal’s natural fur color. Any fur color other than white will render this method practically useless, because it will not stain properly. Another possible limitation is the total number of 4 animals used for behavioral screening in one arena. The explicit number of 4 comes from the fact that the more subjects there are for tracking, the bigger the chance of partial overlap of colors on the visual spectrum. In those cases, absolute separation of colors into separate tracking layers becomes more difficult or even impossible. Red, green, blue, and yellow are quite easy for the camera to separate. Blue and indigo might not be. This tracking method also suffers a bit during night-time recording, as it is very difficult to retain color information under infrared light. Image-processing solutions can be implemented to retrieve color signatures under IR, as each color would have a slightly different shade of gray (Kim et al., 2015). However, that step can be excluded completely when recording is carried out in regular light, but in very dim conditions (e.g., 2 lux). The advancement in image sensor technology is currently high enough to allow for digital exposure boosting, sufficient to visualize colors. Equipping the camera with a bright, prime lens (e.g., with an f-stop value of 1.8) will be of great help here, maximizing the amount of light arriving at the sensor. Digital exposure will undoubtedly introduce some graininess to the final image, however, it will not affect tracking efficacy. Even if the researcher decides to use pose estimation programs for further processing of the video, most are well designed to handle such impediments with their augmentation step.

#### 2.4.3 Advantages and recommendations

Color-based recognition is fantastic when there are no more than 4 subjects to be tracked and the fur color is white. White fur accepts color dyes incredibly easily. Any CD1, FVB, or Balb\c mice, as well as Wistar and Sprague–Dawley rats, are well suited for color-based tracking. When applying dyes to animals, it is important to do so thoroughly (with no “blanks” in between) to ensure the camera recognizes exactly one area of color. Otherwise, a centroid may be miscalculated and will provide slightly incorrect data. It is also a good practice to color a sufficient body area that will be exposed to a camera at all times during various behavioral events (e.g., during rearing behavior when observed from a top-down perspective). Failing to do so may result in a temporary loss of detection area and thus a loss of tracking data. Although paint can be applied to a conscious animal, especially if it is well accustomed to a person handling it, it is advised to perform dyeing under anesthesia to let the paint dry and to avoid the animal licking it while it is still wet. It may also be necessary to re-apply a color when a designated study is long-term and includes particular subjects for multiple testing at different time points. It would be reasonable to test how long a dye stays on the fur before it starts to fade away (using a color-tracking layer for that is highly recommended). Intuitively, dyes intended for coating should not contain any harmful chemicals (i.e., ammonia, peroxide) that may cause adverse effects, such as fur degradation or skin irritation. Any commercial hair dye that lacks harsh chemicals will be an adequate solution. Alternatively, colored pen markers frequently available in animal facilities can be used as well.

## 3 Discussion

It is rather hard to argue with the fact that there is currently no single ideal solution for continuous and uninterrupted subject tracking in multi-animal, behavioral studies. The choice of whether one method or the other is best suited for a particular case will depend on numerous factors and personal preference. Nonetheless, there are a couple of points worth mentioning here to help choose between the options (Table 1). For instance, if behavioral screening consists of only short-term tests (i.e., up to an hour), it might not be necessary to include additional, external markers for identity tracking at all. The proofreading process within pose estimation software should not be overly time-consuming in this case and will provide great efficacy. For multi-hour and multi-day recordings however, implementing external identity markers is highly recommended. Whenever experimental design involves substantial environmental enrichment, the RFiD-supported tracking may be worth considering, as it does not rely solely on visual discrimination to operate. Increasing the size of a transponder and reader antenna can definitely improve tracking accuracy here. If a heavily enriched environment is not used in the experiments, then any presented method can be utilized. It is worth checking the available literature for the efficiency metrics of the particular tracking method. Parameters such as accuracy, precision, or recall, if available, can provide valuable information on whether one method performs better or worse than others and under what circumstances. For long-term monitoring, it is highly unlikely for any of the methods to achieve perfect efficacy. Just as RFiD tracking requires good communication between transponders and readers, pose estimation, ArUco tags, and color-based recognition require good image quality and uninterrupted visual availability of animals. Tracking failures can potentially happen and can be tolerated if they occur for a brief moment. Short “data gaps” can be estimated from tracking values in adjacent frames using data interpolation methods. Proximal interpolation (applying the closest known and existing value) or linear interpolation (predicting values along the straight line between the nearest known data points before and after the gap) can serve as examples, but they are just a few in a plethora of methods. Longer gaps can require more complicated (i.e., machine learning-based) formulas, which can be challenging for non-mathematicians. These methods are widely described in the literature and can even offer “ready-to-use” packages to handle missing data (Rose et al., 2026; Lepot et al., 2017). In addition, pose estimation tools can propagate wrongfully assigned identities into subsequent frames, leaving no tracking gaps whatsoever. In that case, tracking efficacy must be supported with either proofreading or other methods described in this article. For most of the tracking methods, the number of animals used in a single enclosure can be quite high, but one has to keep in mind that the more animals, the bigger the chance of temporarily losing them in the crowd. Also, with more subjects to detect at a particular instance comes a bigger computational demand. For color tracking, the recommended max number of animals is 4, and they have to have white fur, as already mentioned. C57Bl\6 mice will not be suited however, unless the albino strain is selected or the behavioral screening is performed in dyads with one C57Bl\6 mouse and the other mouse in different coating (it might even be possible to not use any dyes at all in that case). In the end, no matter which subject-tracking method is chosen (alone or in combination with pose estimation for example), adopting frameworks that integrate multiple data streams into one automated pipeline can be desirable for any DIY projects. A solid example is Bonsai-rx (Lopes et al., 2015). It is a free, open-source framework where multiple methods can be programmed into and launched within one interface, ensuring their full synchronization and convenience in controlling behavioral experiments. Because some methods can be initially overwhelming when trying to incorporate them into scientific work (e.g., precise positioning with RFiD), it would be very welcome to receive internal support for them in future updates of popular pose estimation tools like DeepLabCut or SLEAP.ai.

**Table 1:** Overview of automated subject tracking methods.

Features	Markerless (pose estimation-based)	RFiD	ArUco	Color-based
Working principle	Image-based processing	Electromagnetism-based, radio frequency transmission	Image-based processing	Image-based processing
Identity carrier	None	Subcutaneously placed transponder (i.e., in the neck or the abdomen area)	Externaly placed tag/marker (i.e., on a tail)	Suitable color dye/paint (animal has to have white fur)
Max number of animal used	No fixed upper limit	No fixed upper limit	No fixed upper limit	Recommendation of 4 (similar colors might be difficult or even impossible to distinguish)
Suited for long-term monitoring	Not recommended on its own, but possible (can be extremely tedious)	Yes	Yes	Yes
Suited for day-time monitoring	Yes	Yes	Yes	Yes
Suited for night-time monitoring	Yes	Yes	Yes	Suited for extremely dim light (i.e., 2 lux), not recommended for infrared light
Suited for enriched environment	Only if animals are not obscured by additions	Suited for any kind if uninterrupted tag-reader communication is ensured	Only if animals are not obscured by additions	Only if animals are not obscured by additions
Minimal computational requirements	A solid GPU is recommended (at least Nvidia RTX series as of 2026)	Standard all-round PC	Standard all-round PC	Standard all-round PC
Essential hardware components	Camera, PC	Camera, PC, additional tags, antennas, control boards, usb hubs, connection cables	Camera, PC, tags	Camera, PC
Implementation challenges	Related to GPU support, laborious annotation process, various hyperparameters tweaking	Related to essential hardware connections, placement, and complex tracking processing	Related to availabe tracking libraries utilization	Related to simple color tracking introduction
Tracking challenges	Related to identity mismatches (swapping) in “crowdy” situations and tracking losses when animals are obscured	Related to possible tracking losses due to “out-of-range” instances and precise position calculations (if needed)	Related to tracking losses when tags are obscured	Related to tracking losses when applied colors are obscured or fade away
Bonsai-rx support	Yes	Yes (although less straightforward than with other methods)	Yes	Yes
Animal welfare considerations	Performing handling procedure	Performing handling procedure, transponder placement under anesthesia	Performing handling procedure, attaching markers with care	Performing handling procedure, animal (re-)coloring under anesthesia, using fur/skin “friendly” dyes
